# Gene Losses and Homology of the Chloroplast Genomes of *Taxillus* and *Phacellaria* Species

**DOI:** 10.3390/genes14040943

**Published:** 2023-04-19

**Authors:** Liwei Wu, Panhui Fan, Jianguo Zhou, Yonghua Li, Zhichao Xu, Yulin Lin, Yu Wang, Jingyuan Song, Hui Yao

**Affiliations:** 1 Key Lab of Chinese Medicine Resources Conservation, State Administration of Traditional Chinese Medicine of the People’s Republic of China, Institute of Medicinal Plant Development, Chinese Academy of Medical Sciences & Peking Union Medical College, Beijing 100193, China; wuliwei2019@163.com (L.W.); fph199901@163.com (P.F.); jgzhou1316@163.com (J.Z.); linyulin@hotmail.com (Y.L.); ywang@implad.ac.cn (Y.W.); jysong@implad.ac.cn (J.S.); 2 Faculty of Pharmacy, Guangxi University of Chinese Medicine, Nanning 530004, China; liyonghua185@126.com; 3 College of Life Science, Northeast Forestry University, Harbin 150040, China; xuzhichao830@126.com

**Keywords:** *Taxillus*, *Phacellaria*, chloroplast genome, gene losses, homology

## Abstract

Research on the chloroplast genome of parasitic plants is limited. In particular, the homology between the chloroplast genomes of parasitic and hyperparasitic plants has not been reported yet. In this study, three chloroplast genomes of *Taxillus* (*Taxillus chinensis*, *Taxillus delavayi*, and *Taxillus thibetensis*) and one chloroplast genome of *Phacellaria* (*Phacellaria rigidula*) were sequenced and analyzed, among which *T. chinensis* is the host of *P. rigidula*. The chloroplast genomes of the four species were 119,941–138,492 bp in length. Compared with the chloroplast genome of the autotrophic plant *Nicotiana tabacum*, all of the *ndh* genes, three ribosomal protein genes, three tRNA genes and the *infA* gene were lost in the three *Taxillus* species. Meanwhile, in *P. rigidula*, the *trnV-UAC* gene and the *ycf15* gene were lost, and only one *ndh* gene (*ndhB*) existed. The results of homology analysis showed that the homology between *P. rigidula* and its host *T. chinensis* was low, indicating that *P. rigidula* grows on its host *T. chinensis* but they do not share the chloroplast genome. In addition, horizontal gene transfer was not found between *P. rigidula* and its host *T. chinensis*. Several candidate highly variable regions in the chloroplast genomes of *Taxillus* and *Phacellaria* species were selected for species identification study. Phylogenetic analysis revealed that the species of *Taxillus* and *Scurrula* were closely related and supported that *Scurrula* and *Taxillus* should be treated as congeneric, while species in *Phacellaria* had a close relationship with that in *Viscum*.

## 1. Introduction

Chloroplasts originated from ancient invasions by eubacteria through symbiosis [1] and are important organelles with autonomic genetic information in plant cells, and they play a crucial role in photosynthesis, amino acid synthesis, and carbon sequestration. With the advancement of sequencing technology, an increasing number of chloroplast genomes have been further resolved [2,3]. In recent years, the chloroplast genomes have been increasingly used as super-barcodes and molecular markers in species identification [4,5,6], and chloroplast sequences have frequently been utilized for constructing plant phylogenies [6,7,8,9]. The typical chloroplast genome of land plants generally consists of two inverted repeats (IR), one large single-copy (LSC), and one small single-copy (SSC) regions, forming a single circular molecule structure [3]. The gene sequence and content of chloroplast genomes are highly conserved [10].

Parasitic plants partially or completely lose the photosynthetic capacity and sustain themselves by acquiring various nutrients and water from their hosts [11]. These characteristics are represented by gene loss [12,13], pseudogenicity [12], and gene transfer [14] at the level of chloroplast genome. Parasitic plants are intermediates of the transition from autotrophic to heterotrophic, whose chloroplast genome analyses help understand the degradation patterns and mechanisms of the chloroplast genomes in angiosperms. Parasitic plants that have retained photosynthesis are called hemiparasite [15]. *Taxillus* plants are hemiparasitic shrubs that belong to the family Loranthaceae, with 18 species in China and 9 endemic species [16]. Many plants in this genus, such as *T. chinensis*, *Taxillus sutchuenensis*, *T. delavayi*, and *T. thibetensis* have medicinal value. *T. chinensis* and *T. sutchuenensis* are the source of the Chinese medicine “*Taxillus herba*”, and *T. delavayi* has been used as an anti-abortifacient herb in Sichuan Province for a long time, and *T. thibetensis* is used as traditional Chinese herbal medicine for clearing lung heat and inducing diuresis [15,17,18].

Many plants serve as hosts, including some parasitic plants. A plant obligated to parasitize on parasitic plants is termed as hyperparasite [19,20]. Hyperparasitic plants are extremely rare, with few species found in Santalales [21]. In the genus *Phacellaria* (Santalaceae), all species are obligate parasites, mainly on Loranthaceae and preferred on genus *Taxillus* [22]. Thirteen chloroplast genomes of *Taxillus* plants have been analyzed [23,24] but only two chloroplast genomes of *Phacellaria* have been determined [25]. Limited knowledge about the chloroplast genomes of *Phacellaria* plants, especially the relationship with their host *Taxillus* plants, is known.

In this study, three chloroplast genomes of *Taxillus* (*T. chinensis*, *T. delavayi*, and *T. thibetensis*) and one chloroplast genome of *Phacellaria* (*P. rigidula*) were sequenced, among which *T. chinensis* is the host of *P. rigidula*. Then, the homology of the chloroplast genomes of *T. chinensis* and *P. rigidula* was analyzed. Finally, comparative and phylogenetic analyses were conducted. This research was the first to investigate the homology between the chloroplast genomes of hyperparasitic plant and their host parasitic plant. The findings could provide a basis for phylogenetic and plant resource research on parasitic and hyperparasitic plants.

## 2. Materials and Methods

### 2.1. Plant Materials

Fresh leaves of *T. delavayi* and *T. thibetensis* were collected from Lijiang City and Dali City in Yunnan Province, respectively, and *T. chinensis* and *P. rigidula* were collected from Fangchenggang City in Guangxi Province. All samples were identified by Professor Yulin Lin. Voucher specimens were deposited in the herbarium at Institute of Medicinal Plant Development. The fresh leaves were stored at −80 °C.

### 2.2. Total DNA Extraction and Sequencing

Total DNA was extracted using the DNease Plant Mini Kit (Qiagen, Hilden, Germany) method. The concentration of total DNA was detected by a micro spectrophotometer (Nanodrop 2000, USA), and its quality was detected by 1% agarose gel electrophoresis. The DNA of four species was used to generate libraries with an average insert size of 500 bp and sequenced using Illumina Hiseq X following the standard protocol. Approximately 5.8 Gb of raw data from *T. chinensis*, 6.9 Gb of raw data from *T. delavayi*, 6.6 Gb from *T. thibetensis*, and 6.9 Gb of raw data from *P. rigidula* were generated with 150 bp paired-end read lengths.

### 2.3. Assembly and Annotation of Chloroplast Genomes

Low-quality reads of all samples were trimmed by Trimmomatic version 0.39 software [26]. GetOrganelle version 1.7.7.0 [27] and NOVOPlasty version 4.3.1 [28] were used to assemble the chloroplast genomes, and GapCloser version 1.12 software [29] was used to fill gaps. GeSeq [30] and CPGAVAS2 software [31] were used to annotate the sequences initially and correct them manually. tRNAscan-SE version 2.0.11 software [32] was used to annotate the tRNA. The annotation results were checked by CPGView [33]. The NCBI accession numbers of complete chloroplast genome sequences of the four species were OQ509063 (*P. rigidula*), OQ509064 (*T. chinensis*), MH161426 (*T. delavayi*), and MH161427 (*T. thibetensis*).

### 2.4. Structural Analysis

Chloroplast genome maps were drawn using Organellar Genome DRAW [34] and manually edited for accuracy. MEGA version 6.0 [35] was used for GC content calculation. CodonW version 1.4.4 software [36] was adopted to analyze the usage of codon. REPuter software [37] was used to identify the long repetitive sequence of chloroplast genome. The type and number of SSR sites were determined using MISA software [38], and the parameter setting was consistent with that in Wu et al. [39]. In this present study, complete repetitive SSR loci were mainly searched, and cycled or reverse complementary SSRs were considered as the same type.

### 2.5. Genome Comparison and Phylogenetic Analysis

Homology analysis was performed using Sibelia version 3.0.7 [40] and Circos version 0.69.9 [41]. Horizontal gene transfer (HGT) analysis was conducted on the basis of sequence similarity through BLAST and phylogenetic trees in accordance with Li et al. [42]. mVISTA [43], an online tool, was utilized to compare the chloroplast genome sequences of *Phacellaria* species. DnaSP version 6.12.03 [44] was used to calculate the nucleotide diversity values (Pi). MAFFT version 5 software [45] was used to compare the sequences. Maximum likelihood (ML) phylogenetic trees were constructed using the program IQTREE version 2.2.2.3 [46].

## 3. Results and Discussion

### 3.1. Basic Characteristics of the Complete Chloroplast Genomes of Three Taxillus Species and P. rigidula

**Figure 1:**
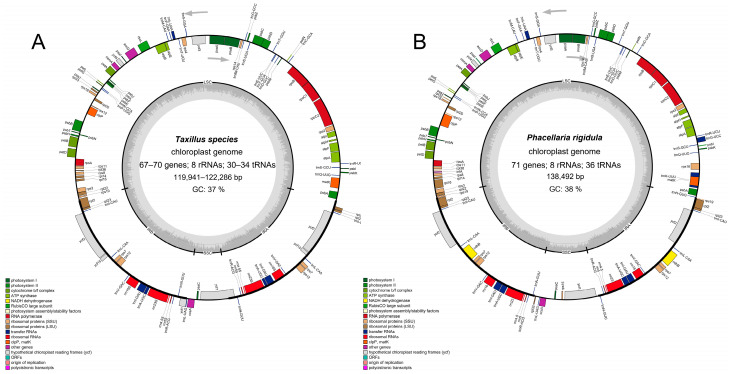
(**A**) Chloroplast genome map of *Taxillus* species, using *T. chinensis* as the template. (**B**) Chloroplast genome map of *P. rigidula*. The grey arrows indicate the direction of transcription of genes. Genes in the inner circle are transcribed in a clockwise direction, while those in the outer circle are transcribed counter-clockwise. The varying shades of gray in the inner circle indicate the distribution of GC and AT content, with darker shades corresponding to GC content and lighter shades corresponding to AT content.

The chloroplast genomes of the three *Taxillus* species and *P. rigidula* all contained an LSC, an SSC, and a pair of IRs, being classical tetrad structures (Figure 1). The chloroplast genomes were 121,363 (*T. chinensis*), 119,941 (*T. delavayi*), 122,286 (*T. thibetensis*), and 138,492 bp (*P. rigidula*) in length. The GC contents in the chloroplast genome of *T. chinensis*, *T. delavayi*, *T. thibetensis*, and *P. rigidula* were 37.3%, 37.1%, 37.2%, and 37.9%, respectively. The GC content was not uniform in the four regions. The IR regions had the highest content (43.0%, 42.4%, 42.7%, and 43.6%), followed by LSC regions (34.7%, 34.7%, 34.5%, and 35.5%) and SSC regions (26.2%, 26.8%, 26.0%, and 29.3%). There were 108 genes annotated in *T. chinensis*, including 67 protein-coding genes, 33 tRNA genes, and 8 rRNA genes. The number of annotated genes in *T. delavayi* was the same as *T. chinensis*, but included 70 protein-coding genes, 30 tRNA genes, and 8 rRNA genes. A total of 112 genes were annotated in *T. thibetensis*, including 70 protein-encoding genes, 34 tRNA genes, and 8 rRNA genes. Meanwhile, in *P. rigidula*, there were 115 genes annotated, including 71 protein-coding genes, 36 tRNA genes, and 8 rRNA genes.

Introns have a significant impact on regulating gene expression, and they can improve the expression of exogenous genes at specific locations in plants, leading to the development of desirable agronomic traits [47]. There were 15 and 12 genes containing introns found in the chloroplast genomes of *P. rigidula* and *T. thibetensis* and 11 genes containing introns found in the chloroplast genomes of *T. chinensis* and *T. delavayi*. The *rps12* gene, a special trans-splicing gene, was similar to some other species [42,48,49].

### 3.2. Gene Losses in the Chloroplast Genomes of Taxillus and Phacellaria Species

The chloroplast genomes of the three *Taxillus* species and *P. rigidula* were compared with an autotrophic plant *N. tabacum* (Z00044), semi-parasitic plant *Viscum minimum* (KJ512176), and total parasitic plant *Epifagus virginiana* (M81884) to investigate the effects of the parasitic lifestyle on the structure and genetic composition of chloroplast genomes.

The chloroplast genome length of parasitic plants was generally shorter, among which *E. virginiana* was the shortest (70,028 bp). The total length of the chloroplast genomes of the three *Taxillus* species was 33,558–35,903 bp shorter than that of *N. tabacum*, and that of *P. rigidula* was 17,352 bp shorter than that of *N. tabacum*. The length of the IR regions of the seven species was relatively similar but the length of the LSC and SSC regions was quite different relatively. Compared with *V. minimum*, which was also a semi-parasitic plant, the LSC regions of the chloroplast genomes of the three *Taxillus* plants were about 5 kb shorter, and the SSC regions were about 3 kb shorter. Meanwhile, the LSC and SSC regions of *P. rigidula* were similar to that of *V. minimum* in length (Supplementary Table S1).

Compared with the chloroplast genome of the autotrophic plant *N. tabacum*, all the *ndh* genes, three ribosomal protein genes (*rps15*, *rps16* and *rpl32*), three tRNA genes (*trnG-UCC*, *trnK-UUU*, and *trnV-UAC*), and the *infA* gene were lost in the chloroplast genomes of *T. chinensis*, *T. delavayi*, and *T. thibetensis*, among which *ndh*, ribosomal protein, and *infA* were genes involved in photosynthesis [50]. In addition, two tRNA genes (*trnA-UGC* and *trnI-GAU*) were lost in *T. delavayi*, and *trnH-GUG* was lost in *T. chinensis*. The *ycf15* gene only appeared in *T. chinensis*. Similar to the chloroplast genomes of *Taxillus* species; *P. rigidula* showed gene losses. Compared with the chloroplast genome of *N. tabacum*, *trnV-UAC,* and *ycf15* were lost in *P. rigidula*, and only one *ndh* gene (*ndhB*) existed in *P. rigidula*. Other studies have also found gene losses in the chloroplast genomes of *Taxillus* and *Phacellaria* species [23,25] (Supplementary Table S2).

### 3.3. Homology Analysis of Chloroplast Genomes of P. rigidula and T. chinensis

**Figure 2:**
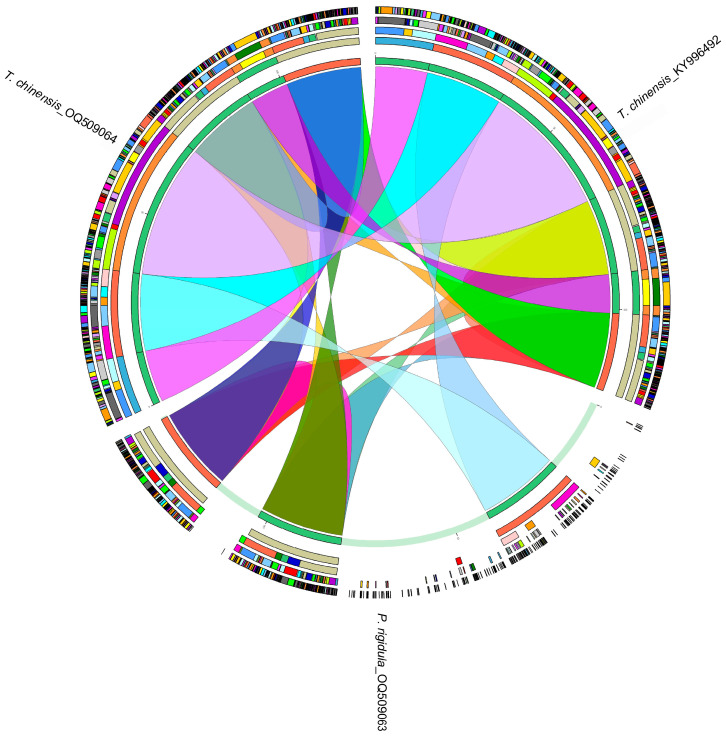
Homology analysis of the chloroplast genomes of *P. rigidula* and *T. chinensis*. The colored blocks and bands represent homologous regions.

Then, the homology of the chloroplast genomes of *P. rigidula*, host *T. chinensis* (OQ509064), and non-host *T. chinensis* (KY996492) were analyzed (Figure 2). The results showed that the homology between the two chloroplast genomes of *T. chinensis* was higher than between *P. rigidula* and its host *T. chinensis* (OQ509064), indicating that *P. rigidula* grows on its host *T. chinensis* (OQ509064), but they do not share the chloroplast genome. *trnA-UGC*, *trnI-GAU*, *trnL-UAA*, and *ycf1* were present in the chloroplast genomes of *P. rigidula* and host *T. chinensis* (OQ509064) but not in non-host *T. chinensis* (KY996492).

HGT, which is the exchange of genes across interspecific barriers, is considered to be a major driver of bacterial evolution [51]. In the present study, all genes in the chloroplast genomes of *P. rigidula* resembled the genes of *Phacellaria* species, and *trnA-UGC*, *trnI-GAU*, *trnL-UAA*, and *ycf1* genes in host *T. chinensis* (OQ509064) resembled the genes of *Tallixus* species, on the basis of sequence similarity through BLAST and phylogenetic trees, indicating HGT was not found between *P. rigidula* and its host *T. chinensis*. HGT is a characteristic of parasitic plants, and it provides strong evidence for direct gene exchange between parasitic plants and host plants [52,53,54]. By using sequence alignment and phylogenetic trees, Li et al. [42] demonstrated that the *rpoC2* gene of *Cistanche deserticola* was transferred from its host plant *Haloxylon ammodendron*. Park et al. [55] also reported HGT events in the *rps2*, *rbcL*, and *trnL-F* genes of *Orobanchaceae* species.

### 3.4. Codon Usage Analysis of the Chloroplast Genomes of Taxillus and Phacellaria Species

Codon usage bias is the non-uniform usage of synonymous codons in the transcripts of proteins, excluding methionine and tryptophan, to encode specific amino acids in the protein [56]. Variation in compositional constraints between genomes is a significant factor in the formation of codon usage bias, as it is influenced by differences in selection pressure and degree of variation [36,57,58]. Furthermore, codon usage bias can be utilized to examine an organism’s evolutionary history, predict expression, and gain insight into the molecular-level evolutionary processes affecting genomes [56,59].

**Figure 3:**
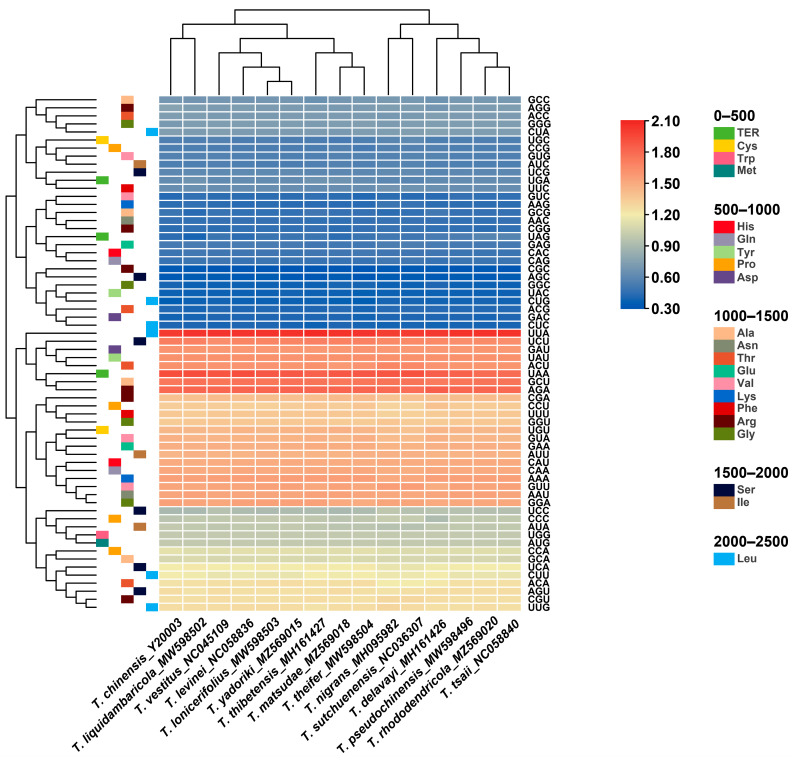
Heat map of RSCU values among *Taxillus* and *Phacellaria* species.

The relative synonymous codon usage (RSCU) of the chloroplast genome of all published *Taxillus* and *Phacellaria* species was calculated on the basis of all protein-coding genes. RSCU measures the frequency of usage of a specific codon relative to the expected frequency and is utilized to identify non-uniform usage of synonymous codons in the coding sequence. Codons with no preference value are assigned a value of 1.00. Codons with an RSCU value > 1.00 are used more frequently than expected, while codons with an RSCU value < 1.00 are used less frequently than expected. The codon usage information of protein-coding genes in the chloroplast genomes of all published *Taxillus* and *Phacellaria* species is shown in Figure 3. A similar codon usage was observed in the chloroplast genomes of *Taxillus* and *Phacellaria* species. These species had 64 codons that code for 20 different amino acids in their chloroplast genomes. In addition, 19,432 (*T. nigrans*)–21,646 (*P. glomerata*) codons were found to encode these amino acids. Among these amino acids, leucine (Leu) was the most widely distributed, whereas cysteine (Cys) was the least distributed. In addition, the codon usage of other amino acids, except for methionine (Met) and tryptophan (Trp), had a preference. Most codons with an RSCU value > 1 were A/U-ending codons, and most codons with an RSCU value ≤ 1 were G/C-ending codons. The chloroplast genome exhibited a greater bias towards the A/U-ending codons than the G/C-ending codons. High codon usage preference, especially high A/U base usage preference, was similar in many plants, such as *Aquilaria sinensis* [60] and *Ulmus* species [61].

GC (the total GC content) reflects the strength of directional mutation pressure, and GC3s (GC content at synonymous third codon position) is closely associated with codon bias and serves as a crucial factor in analyzing codon usage patterns [62,63]. The GC and GC3s values in the codons of the 18 chloroplast genomes examined were less than 0.5, indicating a preference for A/U bases and A/U-ending codons in *Taxillus* and *Phacellaria* species. Analysis of codon adaptation index (CAI) and effective number of codon values (ENc) suggested a minor bias in codon usage among these species, with a relatively low frequency of optimal codons (Fop). Hydrophobicity (Gravy) and aromaticity (Aromo) slightly impacted codon usage bias (Table 1).

### 3.5. Long Repeat Sequences and SSRs

**Figure 4:**
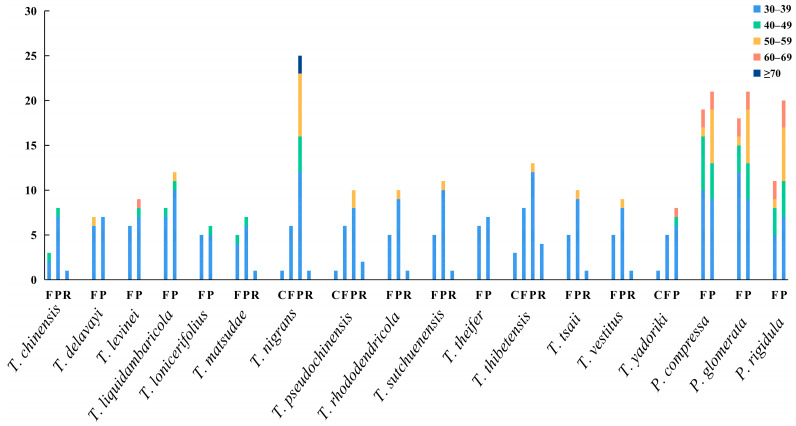
Long repeats of the chloroplast genomes of *Taxillus* and *Phacellaria* species.

Long repeats have significant implications in genome rearrangement and are frequently employed to examine phylogenetic relationships among species. Additionally, long repeats can stimulate intermolecular recombination in chloroplast genomes, thereby enhancing diversity [64]. These long repeats are mainly distributed in the intergene region and intron sequence, including forward (F), palindrome (P), reverse (R), and complement (C). All repeats analyzed in this study were 30 bp or longer and had a sequence similarity of at least 90%. In *Taxillus* species, 11 (*T. lonicerifolius*)–33 (*T. nigrans*) long repeats were identified, most of which were F and P repeats. The length of C and R repeats was mainly within the range of 30–39 bp. Repeats with a length of 60–69 and ≥70 bp only existed in *T. levinei*, *T. nigrans*, and *T. yadoriki*. In *Phacellaria* species, 31 (*P. rigidula*)–40 (*P. compressa*) long repeats were identified, all of which were F and P repeats (Figure 4).

Simple sequence repeats (SSRs), which are also referred to as microsatellite sequences, can be found extensively throughout chloroplast genomes [65]. Due to its high polymorphism, it is increasingly used as molecular markers, species identification, population genetics, and phylogeny [66,67,68]. A total of 45 (*T. levinei*)–87 (*T. thibetensis*) SSRs were detected in the chloroplast genomes of *Taxillus* species, and 40 (*P. compressa*)–43 (*P. rigidula*) SSRs were detected in *Phacellaria* species (Supplementary Tables S3 and S4). The most common mononucleotide repeats were A/T and most dinucleotide repeats were AT/AT. Most SSRs were rich in A/T, which was greatly related to the high A/T content in the chloroplast genome. Moreover, polyA and polyT occupies a relatively high proportion in the SSRs of many plants, compared with polyC and polyG [69].

### 3.6. Comparative Analysis of Chloroplast Genomes

The plant bodies of parasitic plants tend to be simplified; they are morphologically difficult to distinguish [70]. The chloroplast genome has highly variable regions, and these highly variable gene fragments could be used to effectively distinguish congenous species [71]. Here, the highly variable regions in the chloroplast genomes of *Taxillus* and *Phacellaria* species were screened.

**Figure 5:**
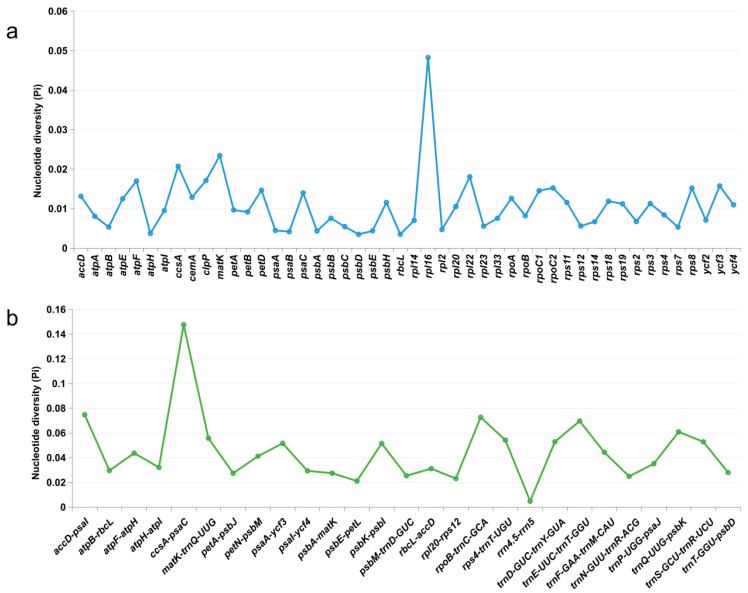
Nucleotide diversity of shared various regions with Pi > 0 and length > 200 bp in 15 chloroplast genomes of *Taxillus* species. (**a**) Pi values in the genes regions. (**b**) Pi values in the intergenic spacers regions.

The nucleotide diversity (Pi) of shared genes and intergenic spacers of the chloroplast genomes of the 15 *Taxillus* species were calculated. In Figure 5, intergenic spacers and genes longer than 200 bp with Pi values greater than 0 are displayed. Polymorphisms were observed to be more frequent in the intergenic spacers (average Pi = 0.0449) compared to the gene regions (average Pi = 0.0108). In accordance with Pi, five candidate highly variable regions were screened out, namely, *ccsA-psaC*, *accD-psaI*, *rpoB-trnC-GCA*, *trnE-UUC-trnT-GGU*, and *rpl16*, for species identification and relationship study.

**Figure 6:**
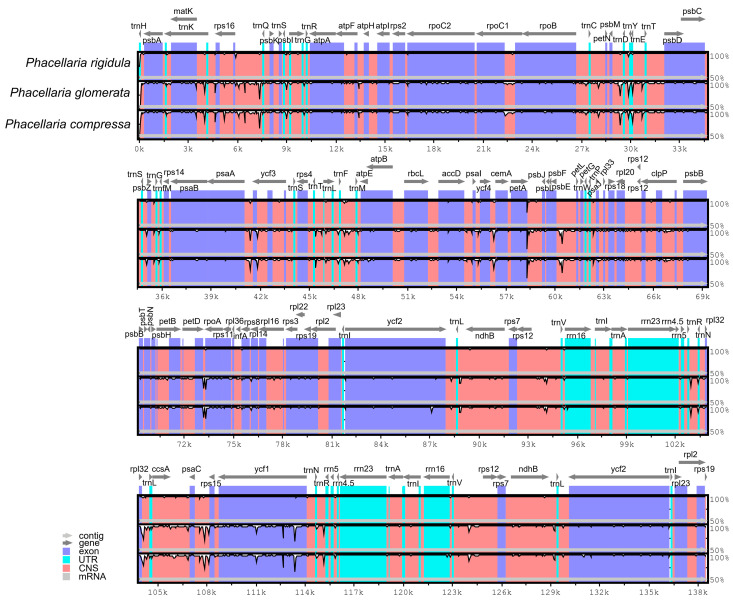
Comparation of chloroplast genomes of 3 *Phacellaria* species. The *x*-axis denotes the positions in the chloroplast genome, while the *y*-axis indicates the average percentage of sequence similarity within the aligned regions, ranging from 50% to 100%.

In addition, with the *P. rigidula* genome as the reference genome, the complete chloroplast genomes of three *Phacellaria* species were compared (Figure 6). The LSC and SSC regions exhibited significantly higher variations compared to the IR regions, while the rRNA genes remained highly conserved with minimal variations. The genes had a very high degree of conservation (most had >90% similarity), and the most varied gene was *ycf1*. Variations in intergenic regions were significantly greater than those in protein-coding regions; these intergenic regions included *rps16-trnQ-UUG*, *psbE-petL*, *rpl32-trnL-UAG*, and *psaC-rps15*.

### 3.7. Phylogenetic Analysis

The relatively conservative chloroplast genome contains a large amount of nucleotide and amino acid sequence information and is of great value in plant evolution, taxonomy, and phylogenetic studies [72]. A phylogenetic tree was constructed using the chloroplast genomes of 50 Santalales species, with *N. tabacum* (Z00044) as the outgroup, to explore the phylogenetic relationship and phylogenetic position of the species in *Taxillus* and *Phacellaria*.

**Figure 7:**
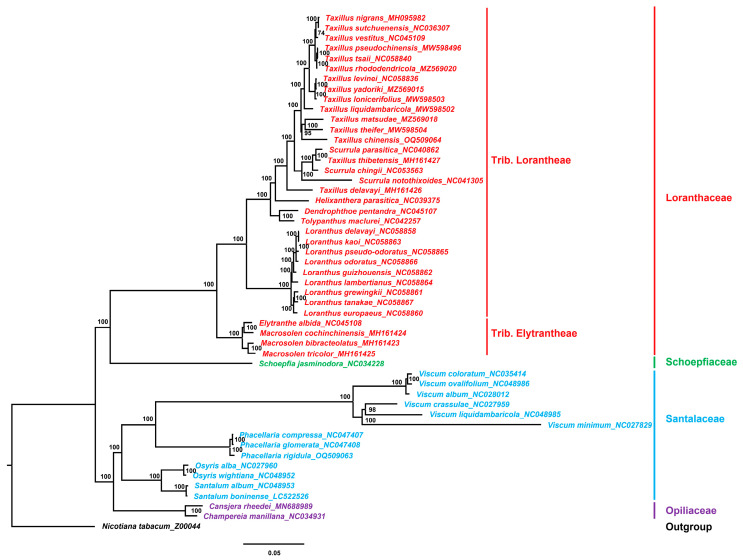
ML phylogenetic tree of 50 Santalales species. Numbers at nodes are bootstrap support values.

The results demonstrated that the bootstrap values were all high (Figure 7). The tree showed that the species of Loranthaceae, Schoepfiaceae, Santalaceae, and Opiliaceae clustered in different branches. In Loranthaceae, the species of Trib. Lorantheae and Trib. Elytrantheae were completely separate. In Trib. Lorantheae, *Taxillus*, *Scurrula*, *Helixanthera*, *Tolypanthus*, and *Dendrophthoe* clustered together, and the species of *Loranthus* clustered in one branch. However, the species of *Taxillus* and *Scurrula* did not form separate branches. In addition, the phylogenetic trees based on common protein-coding genes and chloroplast genes commonly used for phylogenetic analysis (*matK* and *rbcL*) showed that the species in *Taxillus* and *Scurrula* had a close relationship and clustered in one branch (Supplementary Figures S1–S3). Some researchers have suggested that *Scurrula* and *Taxillus* should be treated as congeneric [16]. Pollen morphology studies showed that the pollen of species in *Taxillus* and *Scurrula* was difficult to distinguish, and that species in *Taxillus* and *Scurrula* were closely related [73].

The phylogenetic tree showed that species in *Viscum* and *Phacellaria* clustered together. The classification of *Viscum* plants was always controversial. Loranthaceae used to be divided into Subfam. Loranthoideae and Subfam. Viscoideae, suggesting that *Viscum* belongs to Loranthaceae [74]. However, in the present study, the relationship of the species in Loranthaceae and *Viscum* was far. Previous studies have shown significant differences between species in Loranthaceae and *Viscum* in terms of pollen morphology, chemical composition, and DNA molecular-level; supporting the independent division of Loranthaceae and *Viscum* [73,75]. The results of the present study also supported to divide Loranthaceae and *Viscum* into different families. Some studies divided *Viscum* into Viscaceae family, which is derived from Santalaceae, and they showed that the relationship of species in *Viscum* and Santalaceae was closely related [76]. Meanwhile, in the Angiosperm Phylogeny Group IV system, *Viscum* belongs to Santalaceae. The present study showed that classifying *Viscum* under Santalaceae is more appropriate.

## 4. Conclusions

In this study, three chloroplast genomes of *Taxillus* (*T. chinensis*, *T. delavayi*, and *T. thibetensis*) and one chloroplast genome of *Phacellaria* (*P. rigidula*) were sequenced and analyzed, among which *T. chinensis* is the host of *P. rigidula*. Compared with the chloroplast genome of the autotrophic plant *N. tabacum*, those of *Taxillus* and *Phacellaria* species all showed gene losses, especially the genes involved in photosynthesis. The homology between *P. rigidula* and its host *T. chinensis* was low, indicating that *P. rigidula* grows on its host *T. chinensis* but they do not share the chloroplast genome. In addition, HGT was not found between *P. rigidula* and its host *T. chinensis*. The phylogenetic analysis revealed that the species of *Taxillus* and *Scurrula* were closely related, and supported that *Scurrula* and *Taxillus* should be treated as congeneric. Meanwhile, the species in *Phacellaria* had a close relationship with that in *Viscum*.

## Figures and Tables

**Table 1:** Codon usage of *Taxillus* and *Phacellaria* species.

Species	GC3s	GC	CAI	ENc	Fop	Gravy	Aromo
*T. chinensis*	0.268	0.37	0.165	49.75	0.351	−0.152821	0.116124
*T. delavayi*	0.27	0.372	0.166	49.89	0.354	−0.155567	0.114421
*T. levinei*	0.268	0.371	0.165	49.7	0.351	−0.151184	0.114695
*T. liquidambaricola*	0.269	0.37	0.165	49.75	0.351	−0.152355	0.114921
*T. lonicerifolius*	0.268	0.371	0.165	49.72	0.351	−0.151446	0.114566
*T. matsudae*	0.268	0.37	0.165	49.72	0.352	−0.153982	0.114106
*T. nigrans*	0.276	0.379	0.166	50.10	0.354	−0.133505	0.113285
*T. pseudochinensis*	0.269	0.37	0.165	49.80	0.351	−0.151886	0.115412
*T. rhododendricola*	0.269	0.37	0.166	49.78	0.351	−0.15293	0.115557
*T. sutchuenensis*	0.275	0.379	0.166	50.13	0.354	−0.128203	0.113377
*T. theifer*	0.267	0.37	0.166	49.65	0.351	−0.161496	0.115448
*T. thibetensis*	0.266	0.368	0.166	49.57	0.352	−0.153661	0.115179
*T. tsaii*	0.269	0.37	0.165	49.79	0.351	−0.150685	0.115575
*T. vestitus*	0.269	0.371	0.166	49.84	0.352	−0.158731	0.114805
*T. yadoriki*	0.268	0.37	0.165	49.66	0.351	−0.151572	0.114484
*P. compressa*	0.272	0.381	0.169	50.00	0.356	−0.190492	0.109123
*P. glomerata*	0.273	0.381	0.169	50.02	0.356	−0.192315	0.108927
*P. rigidula*	0.272	0.381	0.169	50.01	0.356	−0.202924	0.108829

## Data Availability

The assembled chloroplast genomes of *P. rigidula*, *T. chinensis*, *T. delavayi* and *T. thibetensis* were deposited in GenBank with the accession numbers OQ509063, OQ509064, MH161426, and MH161427.

## Supplementary Materials

The following supporting information can be downloaded at: https://www.mdpi.com/article/10.3390/genes14040943/s1, Supplementary Table S1: Comparison of the basic information of chloroplast genomes. Supplementary Table S2: Comparison of the gene composition in chloroplast genomes. Supplementary Table S3: Types and amounts of SSRs in the chloroplast genomes of *Taxillus* species. Supplementary Table S4: Types and amounts of SSRs in the chloroplast genomes of *Phacellaria* species. Supplementary Figure S1: Phylogenetic tree constructed using Maximum Likelihood (ML) method based on common protein-coding genes of *Taxillus* and *Scurrula* species. Numbers at nodes are values for bootstrap support. Supplementary Figure S2: Phylogenetic tree constructed using ML method based on *matK* genes of *Taxillus* and *Scurrula* species. Numbers at nodes are values for bootstrap support. Supplementary Figure S3: Phylogenetic tree constructed using ML method based on *rbcL* genes of *Taxillus* and *Scurrula* species. Numbers at nodes are values for bootstrap support.

## References

[1] Dyall S.D., Brown M.T., Johnson P.J. (2004). Ancient invasions: From endosymbionts to organelles. Science.

[2] Clegg M.T., Gaut B.S., Learn G.H., Morton B.R. (1994). Rates and patterns of chloroplast DNA evolution. Proc. Natl. Acad. Sci. USA.

[3] Daniell H., Lin C.S., Yu M., Chang W.J. (2016). Chloroplast genomes: Diversity, evolution, and applications in genetic engineering. Genome Biol..

[4] Li X., Yang Y., Henry R.J., Rossetto M., Wang Y., Chen S. (2015). Plant DNA barcoding: From gene to genome. Biol. Rev. Camb. Philos. Soc..

[5] Song Y., Zhao W., Xu J., Li M., Zhang Y. (2022). Chloroplast genome evolution and species identification of Styrax (Styracaceae). BioMed Res. Int..

[6] Tonti-Filippini J., Nevill P.G., Dixon K., Small I. (2017). What can we do with 1000 plastid genomes?. Plant J..

[7] Lu G., Qiao J., Wang L., Liu H., Wu G., Zhu Y., Zhao Y., Xie G., Qin M. (2022). An integrated study of Violae Herba (*Viola philippica*) and five adulterants by morphology, chemical compositions and chloroplast genomes: Insights into its certified plant origin. Chin. Med..

[8] Feng J.L., Wu L.W., Wang Q., Pan Y.J., Li B.L., Lin Y.L., Yao H. (2022). Comparison analysis based on complete chloroplast genomes and insights into plastid phylogenomic of four Iris species. BioMed Res. Int..

[9] Du Y.P., Bi Y., Yang F.P., Zhang M.F., Chen X.Q., Xue J., Zhang X.H. (2017). Complete chloroplast genome sequences of Lilium: Insights into evolutionary dynamics and phylogenetic analyses. Sci. Rep..

[10] Neuhaus H.E., Emes M.J. (2000). Nonphotosynthetic metabolism in plastids. Annu. Rev. Plant Physiol. Plant Mol. Biol..

[11] Heide-Jørgensen H.S. (2008). Parasitic Flowering Plants.

[12] Petersen G., Cuenca A., Seberg O. (2015). Plastome evolution in hemiparasitic mistletoes. Genome Biol. Evol..

[13] Nie L., Cui Y., Wu L., Zhou J., Xu Z., Li Y., Li X., Wang Y., Yao H. (2019). Gene losses and variations in chloroplast genome of parasitic plant macrosolen and phylogenetic relationships within Santalales. Int. J. Mol. Sci..

[14] Cai L., Arnold B.J., Xi Z., Khost D.E., Patel N., Hartmann C.B., Manickam S., Sasirat S., Nikolov L.A., Mathews S. (2021). Deeply altered genome architecture in the endoparasitic flowering plant *Sapria himalayana* Griff. (Rafflesiaceae). Curr. Biol..

[15] Li Y.H., Ruan J.L., Chen S.L., Lv D., Zhu K.X., Zhao M.H., Pei H.H. (2009). Study on Medicinal Plants of Loranthaceae Resources in China. Mod. Tradit. Chin. Med. Mater. Med. World Sci. Technol..

[16] The Editorial Committee of Flora of China (2003). Flora of China.

[17] Shi X. (2013). Research on Chinese Herbs which Have Diuresis Effect in Chinese Materia Medic. Master’s Thesis.

[18] Li X.M. (2020). The Research on Cognition and Development of the Efficacy of Water-Disinhibiting Damp-Percolating Medicine on the Basis of Ancient and Modern Literature. Master’s Thesis.

[19] Mathiasen R.L., Nickrent D.L., Shaw D.C., Watson D.M. (2008). Mistletoes: Pathology, systematics, ecology, and management. Plant Dis..

[20] Krasylenko Y., Tesitel J., Ceccantini G., Oliveira-da-Silva M., Dvorak V., Steele D., Sosnovsky Y., Piwowarczyk R., Watson D.M., Teixeira-Costa L. (2021). Parasites on parasites: Hyper-, epi-, and autoparasitism among flowering plants. Am. J. Bot..

[21] Wilson C.A., Calvin C.L. (2017). Metadata provide insights on patterns of epiparasitism in mistletoes (Santalales), an overlooked topic in forest biology. Botany.

[22] Li D., Ding Y. (2006). Geographical distribution of *Phacellaria Benth*. (Santalaceae) and its hosts. Front. Biol. China.

[23] Li Y., Zhou J.G., Chen X.L., Cui Y.X., Xu Z.C., Li Y.H., Song J.Y., Duan B.Z., Yao H. (2017). Gene losses and partial deletion of small single-copy regions of the chloroplast genomes of two hemiparasitic *Taxillus* species. Sci. Rep..

[24] Su H.J., Liang S.L., Nickrent D.L. (2021). Plastome variation and phylogeny of *Taxillus* (Loranthaceae). PLoS ONE.

[25] Guo X., Liu C., Wang H., Zhang G., Yan H., Jin L., Su W., Ji Y. (2021). The complete plastomes of two flowering epiparasites (*Phacellaria glomerata* and *P. compressa*): Gene content, organization, and plastome degradation. Genomics.

[26] Bolger A.M., Lohse M., Usadel B. (2014). Trimmomatic: A flexible trimmer for Illumina sequence data. Bioinformatics.

[27] Jin J.J., Yu W.B., Yang J.B., Song Y., de Pamphilis C.W., Yi T.S., Li D.Z. (2020). GetOrganelle: A fast and versatile toolkit for accurate de novo assembly of organelle genomes. Genome Biol..

[28] Dierckxsens N., Mardulyn P., Smits G. (2017). NOVOPlasty: De novo assembly of organelle genomes from whole genome data. Nucleic Acids Res..

[29] Luo R., Liu B., Xie Y., Li Z., Huang W., Yuan J., He G., Chen Y., Pan Q., Liu Y. (2012). SOAPdenovo2: An empirically improved memory-efficient short-read de novo assembler. Gigascience.

[30] Tillich M., Lehwark P., Pellizzer T., Ulbricht-Jones E.S., Fischer A., Bock R., Greiner S. (2017). GeSeq—Versatile and accurate annotation of organelle genomes. Nucleic Acids Res..

[31] Shi L., Chen H., Jiang M., Wang L., Wu X., Huang L., Liu C. (2019). CPGAVAS2, an integrated plastome sequence annotator and analyzer. Nucleic Acids Res..

[32] Schattner P., Brooks A.N., Lowe T.M. (2005). The tRNAscan-SE, snoscan and snoGPS web servers for the detection of tRNAs and snoRNAs. Nucleic Acids Res..

[33] Liu S., Ni Y., Li J., Zhang X., Yang H., Chen H., Liu C. (2023). CPGView: A package for visualizing detailed chloroplast genome structures. Mol. Ecol. Resour..

[34] Lohse M., Drechsel O., Bock R. (2007). OrganellarGenomeDRAW (OGDRAW): A tool for the easy generation of high-quality custom graphical maps of plastid and mitochondrial genomes. Curr. Genet..

[35] Tamura K., Stecher G., Peterson D., Filipski A., Kumar S. (2013). MEGA6: Molecular evolutionary genetics analysis version 6.0. Mol. Biol. Evol..

[36] Sharp P.M., Li W.H. (1987). The codon Adaptation Index—A measure of directional synonymous codon usage bias, and its potential applications. Nucleic Acids Res..

[37] Kurtz S., Choudhuri J.V., Ohlebusch E., Schleiermacher C., Stoye J., Giegerich R. (2001). REPuter: The manifold applications of repeat analysis on a genomic scale. Nucleic Acids Res..

[38] Beier S., Thiel T., Münch T., Scholz U., Mascher M. (2017). MISA-web: A web server for microsatellite prediction. Bioinformatics.

[39] Wu L., Cui Y., Wang Q., Xu Z., Wang Y., Lin Y., Song J., Yao H. (2021). Identification and phylogenetic analysis of five *Crataegus* species (Rosaceae) based on complete chloroplast genomes. Planta.

[40] Minkin I., Patel A., Kolmogorov M., Vyahhi N., Pham S. (2013). Sibelia: A Scalable and Comprehensive Synteny Block Generation Tool for Closely Related Microbial Genomes.

[41] Krzywinski M., Schein J., Birol I., Connors J., Gascoyne R., Horsman D., Jones S.J., Marra M.A. (2009). Circos: An information aesthetic for comparative genomics. Genome Res..

[42] Li X., Zhang T.C., Qiao Q., Ren Z., Zhao J., Yonezawa T., Hasegawa M., Crabbe M.J., Li J., Zhong Y. (2013). Complete chloroplast genome sequence of holoparasite *Cistanche deserticola* (Orobanchaceae) reveals gene loss and horizontal gene transfer from its host *Haloxylon ammodendron* (Chenopodiaceae). PLoS ONE.

[43] Frazer K.A., Pachter L., Poliakov A., Rubin E.M., Dubchak I. (2004). VISTA: Computational tools for comparative genomics. Nucleic Acids Res..

[44] Librado P., Rozas J. (2009). DnaSP v5: A software for comprehensive analysis of DNA polymorphism data. Bioinformatics.

[45] Katoh K., Standley D.M. (2013). MAFFT multiple sequence alignment software version 7: Improvements in performance and usability. Mol. Biol. Evol..

[46] Nguyen L.T., Schmidt H.A., von Haeseler A., Minh B.Q. (2015). IQ-TREE: A fast and effective stochastic algorithm for estimating maximum-likelihood phylogenies. Mol. Biol. Evol..

[47] Xu J., Feng D., Song G., Wei X., Chen L., Wu X., Li X., Zhu Z. (2003). The first intron of rice EPSP synthase enhances expression of foreign gene. Sci. China. Ser. C Life Sci..

[48] Kim K.J., Lee H.L. (2004). Complete chloroplast genome sequences from Korean ginseng (*Panax schinseng* Nees) and comparative analysis of sequence evolution among 17 vascular plants. DNA Res..

[49] Samigullin T.H., Logacheva M.D., Penin A.A., Vallejo-Roman C.M. (2016). Complete plastid genome of the recent holoparasite *Lathraea squamaria* reveals earliest stages of plastome reduction in Orobanchaceae. PLoS ONE.

[50] Li X., Hu Z., Lin X., Li Q., Gao H., Luo G., Chen S. (2012). High-throughput pyrosequencing of the complete chloroplast genome of Magnolia officinalis and its application in species identification. Acta Pharm. Sin..

[51] Bergthorsson U., Adams K.L., Thomason B., Palmer J.D. (2003). Widespread horizontal transfer of mitochondrial genes in flowering plants. Nature.

[52] Davis C.C., Wurdack K.J. (2004). Host-to-parasite gene transfer in flowering plants: Phylogenetic evidence from Malpighiales. Science.

[53] Davis C.C., Anderson W.R., Wurdack K.J. (2005). Gene transfer from a parasitic flowering plant to a fern. Proc. Biol. Sci..

[54] Mower J.P., Stefanović S., Young G.J., Palmer J.D. (2004). Plant genetics: Gene transfer from parasitic to host plants. Nature.

[55] Park J.M., Manen J.F., Schneeweiss G.M. (2007). Horizontal gene transfer of a plastid gene in the non-photosynthetic flowering plants Orobanche and Phelipanche (Orobanchaceae). Mol. Phylogenetics Evol..

[56] Jia J., Xue Q. (2009). Codon usage biases of transposable elements and host nuclear genes in *Arabidopsis thaliana* and *Oryza sativa*. Genom. Proteom. Bioinform..

[57] Paul P., Malakar A.K., Chakraborty S. (2018). Codon usage and amino acid usage influence genes expression level. Genetica.

[58] Hershberg R., Petrov D.A. (2008). Selection on codon bias. Annu. Rev. Genet..

[59] Leffler E.M., Bullaughey K., Matute D.R., Meyer W.K., Ségurel L., Venkat A., Andolfatto P., Przeworski M. (2012). Revisiting an old riddle: What determines genetic diversity levels within species?. PLoS Biol..

[60] Wang Y., Zhan D.F., Jia X., Mei W.L., Dai H.F., Chen X.T., Peng S.Q. (2016). Complete chloroplast genome sequence of *Aquilaria sinensis* (Lour.) gilg and evolution analysis within the Malvales order. Front. Plant Sci..

[61] Zuo L.H., Shang A.Q., Zhang S., Yu X.Y., Ren Y.C., Yang M.S., Wang J.M. (2017). The first complete chloroplast genome sequences of Ulmus species by de novo sequencing: Genome comparative and taxonomic position analysis. PLoS ONE.

[62] Zhao Y., Liu Z., Yang P., Cheng Y., Yang Y. (2016). Codon bias analysis method and research progress on codon bias in *Camellia sinensis*. J. Tea Commun..

[63] Shang M., Liu F., Hua J., Wang K. (2011). Analysis on codon usage of chloroplast genome of *Gossypium hirsutum*. Sci. Agric. Sin..

[64] Park I., Yang S., Choi G., Kim W.J., Moon B.C. (2017). The complete chloroplast genome sequences of *Aconitum pseudolaeve* and *Aconitum longecassidatum*, and development of molecular markers for distinguishing species in the *Aconitum* Subgenus *Lycoctonum*. Molecules.

[65] Powell W., Morgante M., McDevitt R., Vendramin G.G., Rafalski J.A. (1995). Polymorphic simple sequence repeat regions in chloroplast genomes: Applications to the population genetics of pines. Proc. Natl. Acad. Sci. USA.

[66] Yang A.H., Zhang J.J., Yao X.H., Huang H.W. (2011). Chloroplast microsatellite markers in *Liriodendron tulipifera* (Magnoliaceae) and cross-species amplification in *L. chinense*. Am. J. Bot..

[67] Jiao Y., Jia H.M., Li X.W., Chai M.L., Jia H.J., Chen Z., Wang G.Y., Chai C.Y., van de Weg E., Gao Z.S. (2012). Development of simple sequence repeat (SSR) markers from a genome survey of Chinese bayberry (*Myrica rubra*). BMC Genom..

[68] Xue J., Wang S., Zhou S.L. (2012). Polymorphic chloroplast microsatellite loci in Nelumbo (Nelumbonaceae). Am. J. Bot..

[69] Kuang D.Y., Wu H., Wang Y.L., Gao L.M., Zhang S.Z., Lu L. (2011). Complete chloroplast genome sequence of *Magnolia kwangsiensis* (Magnoliaceae): Implication for DNA barcoding and population genetics. Genome.

[70] Clarke C.R., Timko M.P., Yoder J.I., Axtell M.J., Westwood J.H. (2019). Molecular Dialog Between Parasitic Plants and Their Hosts. Annu. Rev. Phytopathol..

[71] Dong W., Liu J., Yu J., Wang L., Zhou S. (2012). Highly variable chloroplast markers for evaluating plant phylogeny at low taxonomic levels and for DNA barcoding. PLoS ONE.

[72] Zhang Y., Li D. (2011). Advances in phylogenomics based on complete chloroplast genomes. Plant Divers. Resour..

[73] Liu L., Qiu H. (1993). Pollen morphology of Loranthaceae in China. Guihaia.

[74] The Editorial Committee of Flora Reipublicae Popularis Sinicae (1988). Flora Reipublicae Popularis Sinicae.

[75] Gong Z., Wang Y., Liang Q., Wang Z., Xu L., Xu G. (2004). A chemotaxonomic study of 27 species of the Loranthaceae plant from China. Guihaia.

[76] Han R., Hao G., Zhang D. (2004). Interfamilial relationships of Santalales as revealed by chloroplast trnL intron sequences. J. Trop. Subtrop. Bot..

