# Supplementary material for: Gene Losses and Homology of the Chloroplast Genomes of Taxillus and Phacellaria Species
Source: Genes (Basel). 2023 Apr 19;14(4):943. doi: 10.3390/genes14040943 (PMC10137875; doi:10.3390/genes14040943)
Supplement: Supplementary file 1 [file genes-14-00943-s001.zip › genes-2341047-supplementary.pdf]

Supplementary Table S1. Comparison of the basic information of chloroplast genomes

Species	*T. chinensis*	*T. delavayi*	*T. thibetensis*	*P. rigidula*	*Viscum minimum*	*Epifagus virginiana*	*Nicotiana tabacum*
Family	Loranthaceae	Loranthaceae	Loranthaceae	Santalaceae	Viscaceae	Orobanchaceae	Solanaceae
Accesion No.	OQ509064	MH161426	MH161427	OQ509063	KJ512176	M81884	Z00044
Genome size(bp)	121,363	119,941	122,286	138,492	131,016	70,028	155,844
LSC length(bp)	70,358	70,281	70,018	79,584	75,814	19,799	86,684
SSC length(bp)	6,077	5,942	6,120	10,652	9,014	4,759	18,482
IRa length(bp)	22,464	21,859	23,074	24,128	23,094	22,735	25,339
IRb length(bp)	22,464	21,859	23,074	24,128	23,094	22,735	25,339
GC content(%)	37.3	37.1	37.2	37.9	36.2	37.5	37.8
Number of genes	108	108	112	115	104	53	151
Number of protein-coding genes	67	68	67	71	66	10	112
Number of tRNAs	33	30	34	36	29	17	30
Number of rRNAs	8	8	8	8	8	8	8

Supplementary Table S2. Comparison of the gene composition in chloroplast genomes

Genes	*T. chinensis*	*T. delavayi*	*T. thibetensis*	*P. rigidula*	*Viscum minimum*	*Epifagus virginiana*	*Nicotiana tabacum*
accD	+	+	+	+	+	+	+
atpA	+	+	+	+	+	+	+
atpB	+	+	+	+	+	+	+
atpE	+	+	+	+	+	-	+
atpF	+	+	+	+	+	-	+
atpH	+	+	+	+	+	-	+
atpI	+	+	+	+	+	-	+
ccsA (ycf5)	+	+	+	+	+	-	+
cemA (ycf10)	+	+	+	+	+	-	+
clpP	+	+	+	+	+	+	+
infA	-	-	-	+	-	+	+
matK	+	+	+	+	+	+	+
ndhA	-	-	-	-	-	-	+
ndhB	-	-	-	+	+	+	+
ndhC	-	-	-	-	-	-	+
ndhD	-	-	-	-	-	-	+
ndhE	-	-	-	-	-	-	+
ndhF	-	-	-	-	-	-	+
ndhG	-	-	-	-	-	-	+
ndhH	-	-	-	-	-	-	+
ndhI	-	-	-	-	-	-	+
ndhJ	-	-	-	-	-	-	+
ndhK	-	-	-	-	-	-	+
petA	+	+	+	+	+	-	+
petB	+	+	+	+	+	-	+
petD	+	+	+	+	+	-	+
petG	+	+	+	+	+	-	+
petL	+	+	+	+	+	-	+
petN	+	+	+	+	+	-	-
psaA	+	+	+	+	+	-	+
psaB	+	+	+	+	+	-	+
psaC	+	+	+	+	+	-	+
psaI	+	+	+	+	+	-	+
psaJ	+	+	+	+	+	-	+
psbA	+	+	+	+	+	+	+
psbB	+	+	+	+	+	+	+
psbC	+	+	+	+	+	-	+
psbD	+	+	+	+	+	-	+
psbE	+	+	+	+	+	-	+
psbF	+	+	+	+	+	-	+

psbH	+	+	+	+	+	-	+
psbI	+	+	+	+	+	-	+
psbJ	+	+	+	+	+	-	+
psbK	+	+	+	+	+	-	+
psbL	+	+	+	+	+	-	+
psbM	+	+	+	+	+	-	+
psbN	+	+	+	+	+	-	+
psbT	+	+	+	+	+	-	+
psbZ (ycf9)	+	+	+	+	+	-	+
rbcL	+	+	+	+	+	+	+
rpl12	-	-	-	-	-	+	-
rpl14	+	+	+	+	+	+	+
rpl16	+	+	+	+	+	+	+
rpl2	+	+	+	+	+	+	+
rpl20	+	+	+	+	+	+	+
rpl22	+	+	+	+	+	-	+
rpl23	+	+	+	+	+	+	+
rpl32	-	-	-	+	+	-	+
rpl33	+	+	+	+	-	+	+
rpl36	+	+	+	+	+	+	+
rpoA	+	+	+	+	+	+	+
rpoB	+	+	+	+	+	-	+
rpoC1	+	+	+	+	+	-	+
rpoC2	+	+	+	+	+	-	+
rps11	+	+	+	+	+	+	+
rps12	+	+	+	+	+	+	+
rps14	+	+	+	+	+	+	+
rps15	-	-	-	+	+	-	+
rps16	-	-	-	+	+	-	+
rps18	+	+	+	+	+	+	+
rps19	+	+	+	+	+	+	+
rps2	+	+	+	+	+	+	+
rps3	+	+	+	+	+	+	+
rps4	+	+	+	+	+	+	+
rps7	+	+	+	+	+	+	+
rps8	+	+	+	+	+	+	+
rrn16S	+	+	+	+	+	+	+
rrn23S	+	+	+	+	+	+	+
rrn4.5S	+	+	+	+	+	+	+
rrn5S	+	+	+	+	+	+	+
trnA-UGC	+	-	+	+	+	+	+
trnC-GCA	+	+	+	+	+	+	+
trnD-GUC	+	+	+	+	+	+	+
trnE-UUC	+	+	+	+	+	+	+

trnF-GAA	+	+	+	+	+	+	+
trnfM-CAU	+	+	+	+	+	-	+
trnG-GCC	+	+	+	+	+	-	+
trnG-UCC	-	-	-	+	+	-	+
trnH-GUG	-	+	+	+	+	+	+
trnI-CAU	+	+	+	+	-	+	-
trnI-GAU	+	-	+	+	+	+	+
trnK-UUU	-	-	-	+	+	-	+
trnL-CAA	+	+	+	+	+	+	+
trnL-UAA	+	+	+	+	+	-	+
trnL-UAG	+	+	+	+	+	+	+
trnM-CAU	+	+	+	+	+	+	+
trnN-GUU	+	+	+	+	+	+	+
trnP-UGG	+	+	+	+	+	+	+
trnQ-UUG	+	+	+	+	+	+	+
trnR-ACG	+	+	+	+	+	+	+
trnR-UCU	+	+	+	+	+	+	+
trnS-GCU	+	+	+	+	+	+	+
trnS-GGA	+	+	+	+	+	+	+
trnS-UGA	+	+	+	+	+	+	+
trnT-GGU	+	+	+	+	+	-	+
trnT-UGU	+	+	+	+	+	-	+
trnV-GAC	+	+	+	+	+	-	+
trnV-UAC	-	-	-	-	-	-	+
trnW-CCA	+	+	+	+	+	+	+
trnY-GUA	+	+	+	+	+	+	+
ycf1	+	+	+	+	-	-	+
ycf2	+	+	+	+	+	+	+
ycf3	+	+	+	+	+	-	+
ycf4	+	+	+	+	+	-	+
ycf15	+	-	-	-	-	-	+

（+: present;　-: missing）

Supplementary Table S3. Types and amounts of SSRs in the chloroplast genomes of *Taxillus* species

SSR type	Repeat unit	*T. chinensis*	*T. delavayi*	*T. levinei*	*T. liquidambaricola*	*T. lonicerifolius*	*T. matsudae*	*T. nigrans*	*T. pseudochinensis*	*T. rhododendricola*	*T. sutchuenensis*	*T. theifer*	*T. thibetensis*	*T. tsaii*	*T. vestitus*	*T. yadoriki*
Mono	A/T	45	49	32	40	36	37	40	42	36	38	41	55	44	44	36
	C/G	3	1	-	4	-	2	1	1	-	1	2	1	1	2	-
Di	AC/GT	-	-	-	-	-	-	-	-	-	-	1	-	-	-	-
	AG/CT	-	-	-	-	-	2	-	-	-	-	-	3	-	-	-
	AT/AT	6	8	5	8	5	11	12	11	9	10	7	18	11	7	4
Tri	AAG/CTT	2	2	2	2	2	2	2	2	2	2	2	2	2	2	2
	AAT/ATT	-	3	-	1	-	-	2	1	1	2	-	1	1	2	-
Tetra	AAAG/CTTT	1	1	2	1	2	-	-	-	-	-	-	3	-	-	2
	AAAT/ATTT	-	-	1	2	-	1	-	-	-	-	-	1	-	-	2
	AAGG/CCTT	-	-	-	1	-	-	-	-	-	-	-	-	-	-	-
	AAGT/ACTT	-	-	1	1	1	-	1	1	1	1	-	-	1	1	-
	AATC/ATTG	1	-	-	-	-	-	-	-	-	-	-	-	-	-	-
	AATG/ATTC	-	-	-	-	-	-	-	-	-	-	-	-	2	-	-
	AATT/AATT	-	-	1	1	1	1	1	1	1	-	1	-	1	3	-
	ACAG/CTGT	1	1	1	1	1	1	1	1	1	1	1	-	1	1	1
	ACAT/ATGT	-	-	-	-	-	-	-	1	1	-	-	-	1	-	-
	AGAT/ATCT	-	-	-	-	2	-	-	-	-	-	-	-	-	2	2
	AGGG/CCCT	-	2	-	-	-	-	-	-	-	-	-	2	-	-	-
Penta	AAAAT/ATTTT	-	1	-	-	-	-	-	-	-	-	-	-	-	-	-
	AATAT/ATATT	1	-	-	-	-	1	1	1	1	1	-	-	1	1	-
	ACTAT/AGTAT	-	-	-	-	-	-	-	-	-	-	-	-	-	1	-
	ATATC/ATATG	-	-	-	-	-	-	-	-	-	-	-	1	-	-	-

Supplementary Table S4. Types and amounts of SSRs in the chloroplast genomes of *Phacellaria* species

SSR type	Repeat unit	*P. rigidula*	*P. compressa*	*P. glomerata*
Mono	A/T	31	28	30
	C/G	1	1	1
Di	AG/CT	2	2	2
	AT/AT	2	4	4
Tri	AAT/ATT	2	2	2
	AAC/GTT	-	1	
Tetra	AAAT/ATTT	-	1	2
	AATC/ATTG	2	-	-
	ACAG/CTGT	1	1	1
Penta	ACTAT/AGTAT	1	-	-
Hexa	AATGAT/ATCATT	1	-	-

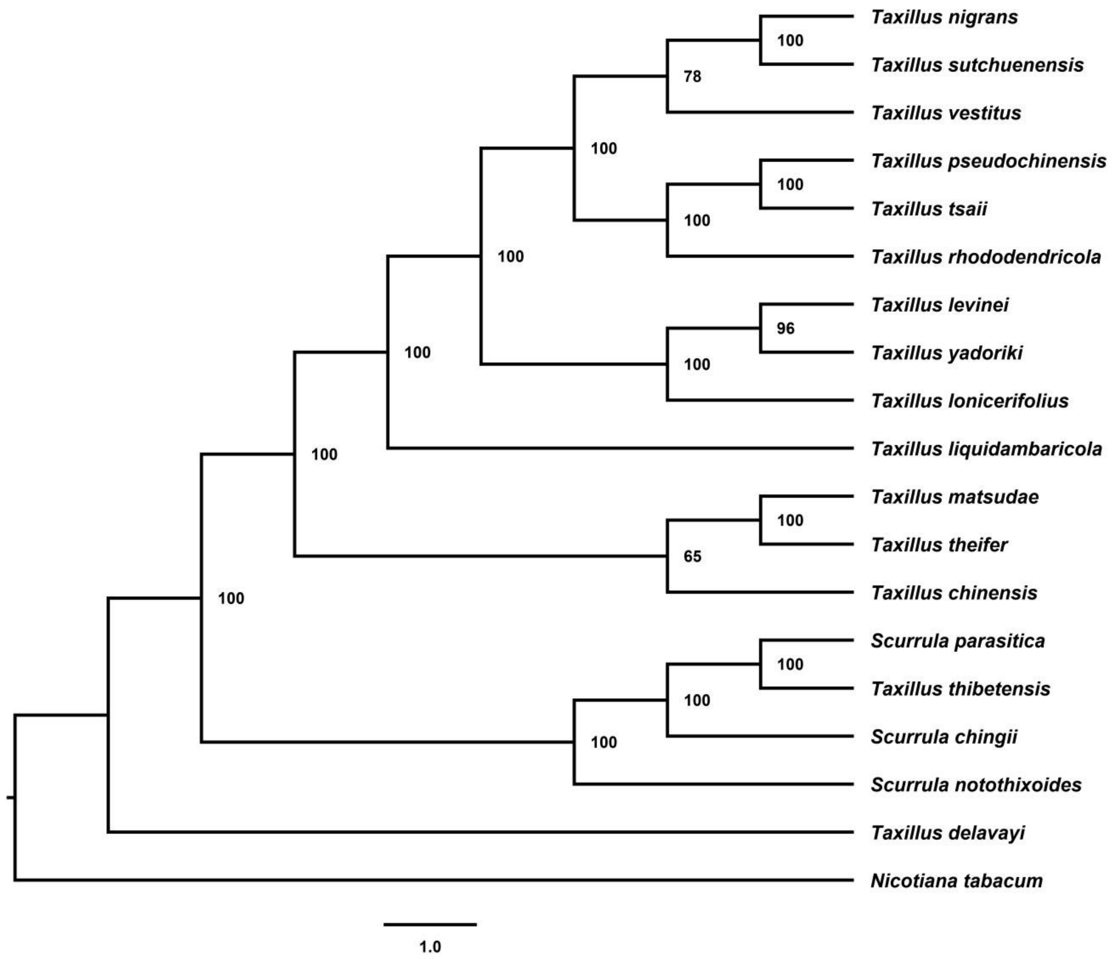

Supplementary Figure S1. Phylogenetic tree constructed using Maximum Likelihood (ML) method based on common protein-coding genes of *Taxillus* and *Scurrula* species. Numbers at nodes are values for bootstrap support.

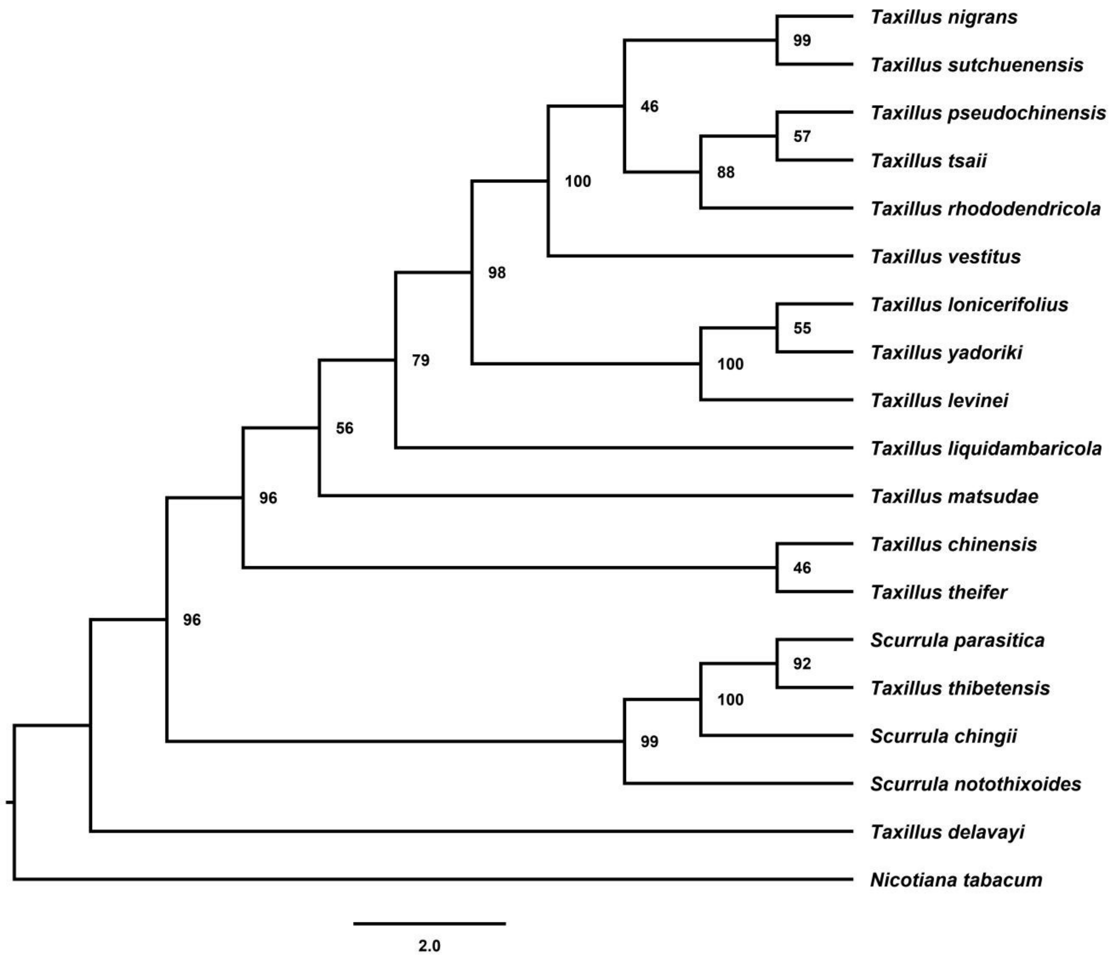

Supplementary Figure S2. Phylogenetic tree constructed using ML method based on *matK* genes of *Taxillus* and *Scurrula* species. Numbers at nodes are values for bootstrap support.

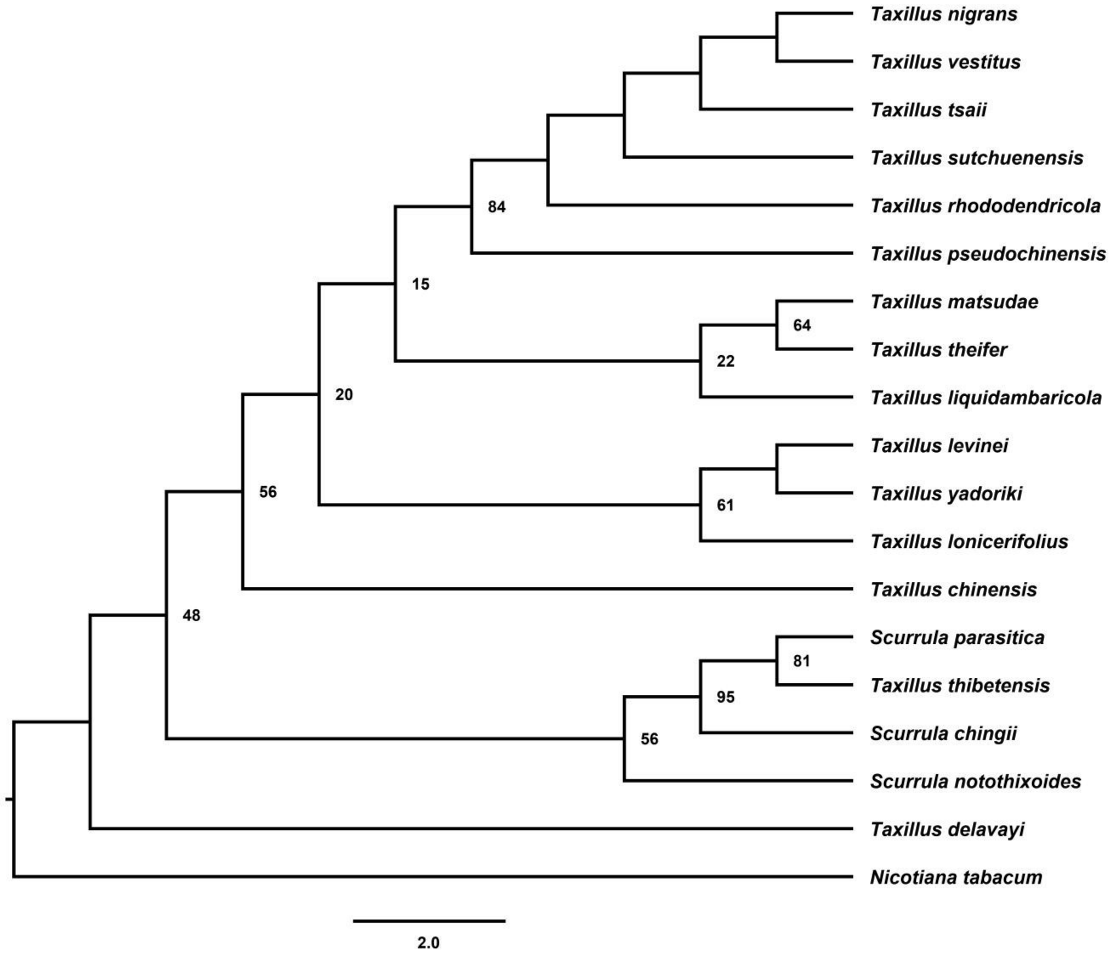

Supplementary Figure S3. Phylogenetic tree constructed using ML method based on *rbcL* genes of *Taxillus* and *Scurrula* species. Numbers at nodes are values for bootstrap support.
